# Neurophysiological Characteristics Associated with Driving Abilities in Older Adults: A Scoping Review

**DOI:** 10.3390/jcm15082956

**Published:** 2026-04-13

**Authors:** Mutsuhide Tanaka, Yuma Hidaka, Futoshi Mori

**Affiliations:** 1 Department of Occupational Therapy, Faculty of Health Science, Prefectural University of Hiroshima, 1-1 Gakuen-cho, Mihara-shi 723-0053, Hiroshima, Japan; mtanaka@pu-hiroshima.ac.jp; 2 Doctoral Program of Clinical Neuropsychiatry, Graduate School of Health Science, Kagoshima University, 8-35-1, Sakuragaoka, Kagoshima-shi 890-8544, Kagoshima, Japan; k0745655@kadai.jp; 3 Department of Rehabilitation, Medical Corporation, Sanshukai, Okatsu Hospital, 3-9, Masagohonmachi, Kagoshima-shi 890-0065, Kagoshima, Japan

**Keywords:** driving ability, neurophysiology, older adults, cognitive decline, fitness-to-drive assessment, electroencephalography, midfrontal theta, prefrontal cortex, driving simulator, event-related potentials

## Abstract

With population aging, motor vehicle accidents involving older drivers have increased. Age-related cognitive decline affects driving performance; however, the underlying neural mechanisms remain unclear. This scoping review explored neurophysiological characteristics associated with driving in older adults, including those at risk of dementia. Following PRISMA-ScR guidelines, we searched PubMed, Scopus, and CINAHL for studies examining driving-related neurophysiological measures in older adults aged ≥60 years. Twelve studies were included. Findings converge on load-dependent neural compensation failure: older adults maintain driving performance under low-to-moderate demands, but compensatory mechanisms break down under high cognitive load. Electroencephalography (EEG) studies revealed blunted midfrontal theta upregulation during high-load conditions, associated with reduced steering precision and delayed responses. Event-related potential studies demonstrated that reduced P3b amplitude was associated with missed braking responses and that abnormal visual evoked potentials in Alzheimer’s disease predicted unfit-to-drive classifications. fNIRS studies during driving-related tasks and an fMRI study using a laboratory-based visual task consistently showed prefrontal hyperactivation in older adults. Although some older adults maintained comparable performance to younger adults, the brain–behavior associations observed in younger adults were absent, suggesting that this hyperactivation does not necessarily serve a functional compensatory role. Combined with EEG evidence of impaired oscillatory modulation, these findings suggest that prefrontal hyperactivation does not necessarily compensate for diminished neural synchronization under high-load conditions. Neurophysiological markers hold promise for fitness-to-drive assessments. Future research should employ high-load scenarios and multimodal neuroimaging to verify prefrontal compensatory mechanisms.

## 1. Introduction

As the global population of older adults increases, so does the number of older adults who drive [1,2]. Driving is a crucial means of transportation for older adults, particularly because age-related physical decline often reduces their mobility and independence [3,4]. Furthermore, the ability to operate a personal vehicle affords greater convenience than relying on public transport [5,6]. Therefore, driving is essential for older adults to maintain independent living and participate in social activities.

The prevalence of car accidents involving older adults is a significant global concern. Individuals aged ≥75 years have the highest rates of at-fault accidents and injury, irrespective of driving distance [7,8,9]. The incidence of fatal accidents among this demographic remains disproportionately higher than that among younger drivers, even when accounting for the distance traveled [10], as well as the higher risk of sustaining fatal or serious injuries in frontal collisions compared to younger drivers [11]. In addition, the Alzheimer’s Association has reported that approximately 60% of individuals with mild cognitive impairment (MCI) and 30% of individuals with Alzheimer’s disease (AD) continue to drive in the United States [12]. A thorough evaluation of older drivers’ driving capabilities, along with the implementation of advanced driver-assistance systems (ADAS) tailored to their needs, is a pressing societal concern.

Age-related declines in physical and cognitive function contribute to impaired driving performance among older drivers. Older drivers tend to have much slower braking reaction times, influenced by age-related physical declines, such as weakened pedal control from reduced leg muscle strength [13,14], and cognitive declines, including impaired attention, executive function [15,16], and overall cognition [17]. Evidence suggests that accelerator-brake pedal misapplication, delayed response times, and impaired error correction are driven more by deficits in attention and executive function than by reduced lower-limb strength or motor response speed [18,19]. Impairments in attention and executive function are significantly associated with increased steering instability and greater lane-keeping variability [20,21], effects that are particularly pronounced among drivers with MCI [22]. Age-related declines in visuospatial cognition and visual search ability underlie attentional impairments in older drivers [23,24].

Although structural brain changes and dementia-related neuromarkers have been linked to driving risk in older adults [25,26,27,28,29,30,31], these parameters primarily reflect neuropathology and do not directly assess cognitive control during driving. Neurophysiological measurements of brain function provide an objective approach to quantifying age-related cognitive impairment. Electroencephalography (EEG) studies show that age-related alterations in cognitive processes are intricately linked to changes in driving skills and capabilities in older adults. Frontal theta power, which supports cognitive and motor control, mental workload, and sensorimotor integration, declines with aging [32], and its task-dependent modulation is attenuated [33]. In contrast, posterior alpha power, associated with attention allocation, attentional withdrawal, and boredom, also declines with aging and either remains unchanged or exhibits compensatory increases under a high cognitive load [34,35,36]. Functional magnetic resonance imaging (fMRI) studies corroborate these EEG findings, revealing compensatory increases in prefrontal cortex activity and relative decreases in occipital activity under high mental and cognitive loads in older adults [37,38], as well as increased and reorganized functional connectivity between the prefrontal and parietal cortices and within attentional networks [39,40]. These cognitive control-related brain activities characteristic of older adults indicate both compensation for functional brain decline and the limitations of such compensation, as exemplified by attenuated cognitive control and attention resulting from default mode network (DMN) decoupling [37,41].

Previous neurophysiological studies suggest that quantitative and objective measurements of brain activity during driving in older adults have potential applications for driving fitness assessment and ADAS. We hypothesized that driving performance in older drivers is supported by functional changes in brain activity associated with cognitive control and attentional allocation, including prefrontal hyper- or hypoactivation and reduced fronto-parietal connectivity.

This scoping review aimed to synthesize neurophysiological studies of brain activity during real-vehicle and driving-simulator operations in older adults and to elucidate the relationship between the driving performance characteristics of older drivers and the neural processing of associated cognitive functions. Through this synthesis, we aimed to identify evidence gaps between age-related vulnerability in brain function contributing to driving risk and current driving skill assessments and provide implications for the development of neurophysiological evaluation methods and safe driving support technologies.

## 2. Materials and Methods

This scoping review applied the Preferred Reporting Items for Systematic Reviews and Meta-Analyses extension for Scoping Reviews (PRISMA-ScR) framework [42]. The completed PRISMA-ScR checklist is provided in Table S1: PRISMA-ScR Checklist for “Neurophysiological Characteristics Associated with Driving Abilities in Older Adults: A Scoping Review”. Using this framework, we classified and reviewed studies using EEG, fMRI, or other neurophysiological methods to identify patterns related to driving skills, driving capabilities, and relevant cognitive control.

### 2.1. Protocol and Registration

This scoping review was registered on the Open Science Framework (OSF, https://doi.org/10.17605/OSF.IO/KA5G4). The complete protocol, including eligibility criteria and search methods, is publicly accessible.

### 2.2. Research Questions

We defined research questions to clarify the objective of this review and develop the review strategy. The research questions were: 1. How extensively have neurophysiological correlates of driving behavior in older adults been elucidated? 2. What characteristics of resting or task-related brain activity predict a history of crashes, hazardous driving behavior, or criterion of driving cessation in older drivers (such as on-road incidents or driving simulator outcomes? These questions were explored using the PCC (Population–Concept–Context) framework [43]:

- Population: Older adults aged ≥60 years who hold a valid private driving license and drive regularly.
- Concept: Neurophysiological and functional brain characteristics measured during driving or associated with risky driving (including EEG, event-related potentials [ERP], functional MRI, functional near-infrared spectroscopy; fNIRS, positron emission tomography; PET).
- Context: Evaluation of driving safety and prediction of hazardous driving risk.

### 2.3. Inclusion and Exclusion Criteria

Studies were included if they met the following criteria: 1. experimental or observational studies (including cross-sectional and cohort designs) reporting neurophysiological measures associated with driving performance or risk in older adults. Narrative reviews, editorials, commentaries, and case reports were excluded. 2. Human participants aged ≥60 years who hold a driving license and drive regularly. This threshold was chosen because age-related cognitive decline may emerge before age 65 [44,45]. Studies spanning the full adult age range were also eligible provided that neurophysiological data for participants aged ≥60 years were reported separately or stratified by age group. Studies involving participants with dementia or MCI were included if they still drive. 3. Measures of driving ability, driving performance, driving behavior, or crash/near-crash outcomes; studies must report neurophysiological data (EEG, ERP, fMRI, fNIRS, functional PET, cortical activity) collected during driving tasks, driving simulations, or at rest. 4. Publications in English. 5. Published between 2015 and 2025.

### 2.4. Information Sources and Search Strategy

We comprehensively searched and retrieved from the following databases: PubMed, Scopus, and CINAHL. Although Web of Science was not searched due to limited institutional access, Scopus provides broad interdisciplinary coverage and partially overlaps with it. The search was limited to the past decade (2015–2025) because neurophysiological findings, particularly EEG/ERP measures, are highly sensitive to signal processing and analytical methodologies. Standardized time–frequency decomposition methods (e.g., event-related spectral perturbation analysis) and independent component analysis (ICA)-based artifact rejection became widely adopted following the systematization of these techniques [46,47], and automated ICA component classification was further advanced in recent years [48]. Studies published before 2015 typically employed heterogeneous preprocessing pipelines that differ from contemporary standards, rendering direct comparison with recent findings methodologically challenging. Two independent reviewers (M.T. and Y.H.) searched the electronic databases PubMed, CINAHL, and Scopus on 3 August and 6 November 2025, respectively. For PubMed, the following search expression was used and adapted to other databases: ((“Aged”[MeSH] OR elderly OR “older adults” OR “senior” OR aging OR “older driver” OR “geriatric” OR “Mild Cognitive Impairment”[MeSH] OR MCI OR dementia OR “Subjective Cognitive Decline”[tiab] OR “Subjective Memory Complaint”[tiab] OR “Motoric Cognitive Risk Syndrome”[tiab]) AND (“Automobile Driving”[MeSH] OR driving OR “driving ability” OR “driving performance” OR “driving behavior” OR “driving skill” OR “driving competence” OR “driving simulation” OR “driving simulator”) AND (“neurophysiology”[MeSH Terms] OR neurophysiological OR “neural marker*” OR “brain imaging” OR neuroimaging OR “cortical activity” OR “brain activity” OR “brain function” OR “cognitive function” OR electroencephalography OR EEG OR “event-related potential” OR ERP OR “functional magnetic resonance imaging” OR fMRI OR “near infrared spectroscopy” OR fNIRS OR “positron emission tomography” OR PET) AND (“driving safety” OR “traffic accident” OR “crash” OR “collision” OR “road safety” OR hazard OR error OR “car accident” OR “risk assessment” OR attention)) AND (english[lang]).

Gray literature was searched using Google Scholar and OSF preprints. The reference lists of included articles were manually searched, and forward citation tracking was conducted in Google Scholar for all included studies to identify recent articles. Additionally, we also hand-searched potentially relevant preprints and non-index records.

### 2.5. Selection of Sources of Evidence

The search was conducted by two independent reviewers (M.T. and Y.H.). All searched studies were imported into EndNote 21 and then uploaded to Rayyan to delete duplicate studies and screen them. Two reviewers conducted Title/abstract and full-text screening on the Rayyan platform, following the eligibility criteria. Any discrepancies in the selection were solved through online discussion. We asked the third reviewer (F.M.) to review the results at each screening step and consulted in cases of uncertainty.

### 2.6. Data Charting Process

Data extraction was performed independently by two reviewers (M.T. and Y.H.) using a standardized data charting form. Discrepancies were resolved through discussion, with consultation from a third reviewer (F.M.) when necessary. The data charting form was piloted on the first three included studies and refined accordingly.

We extracted the following data: author, title, publication year, population or target population, sample size, study objectives, neurophysiological modality, driving task or context, driving or behavioral outcomes, key neurophysiological findings, and key findings of brain–behavior associations.

### 2.7. Critical Appraisal

Consistent with scoping review methodology, a formal critical appraisal of the included studies was not conducted, as the primary aim was to map the extent and nature of the available evidence rather than to assess study quality [43].

### 2.8. Synthesis of Results

A narrative synthesis approach was employed. Findings were organized thematically by neurophysiological modality and driving assessment type, as presented in Table 1. Brain–behavior associations were synthesized to identify consistent patterns across studies.

## 3. Results

### 3.1. Selection Process

**Figure 1:**
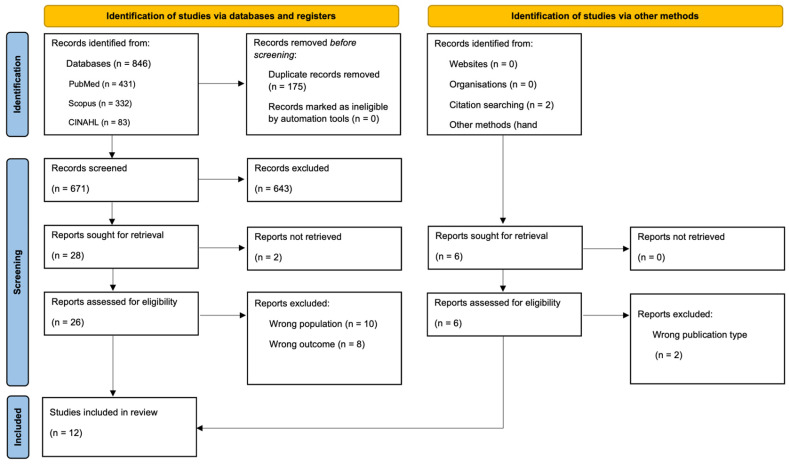
PRISMA flow diagram.

Figure 1 presents the study selection process. A total of 846 records were identified through electronic database searches, including PubMed (*n* = 431), Scopus (*n* = 332), and CINAHL (*n* = 83) databases. An additional 6 records were identified through citation tracking (*n* = 2) and hand searching (*n* = 4). After removing 175 duplicates, 671 records remained for further screening. Title and abstract screening excluded 643 records, and 32 full-text articles (26 from database searches and 6 from other methods) were assessed for their eligibility; 2 reports could not be retrieved. Of these, 20 reports were excluded with reasons, and 12 studies met the inclusion criteria and were included in the qualitative synthesis.

### 3.2. Overview of Included Article

The neurophysiological methods used in the studies included in this scoping review were EEG/ERP in eight studies [49,50,51,52,53,54,55,56], fNIRS in three studies [57,58,59], and fMRI in one study [60] (Table 1). All participants were aged ≥60 years. Nine studies compared neurophysiological indices during simulated driving between older drivers and younger adults [51,52,54,55,56,57,58,59,60], although three studies also examined differences across multiple older age subgroups [49,50,53]. With respect to cognitive function, one study contrasted drivers with AD with cognitively healthy older adults [55], while in other cases, older participants were differentiated into cognitively healthy and subtly cognitively impaired groups to examine age-related cognitive decline [51].

Most studies employed driving simulator tasks [49,50,51,52,53,56,58,59], whereas the remaining four studies used laboratory-based experiments that isolated specific driving-related components: bipedal response tasks [57], acceleration/deceleration visual-stimulus oddball tasks [55], traffic sign recognition tasks [54], and visual speed discrimination tasks [60]. Sample sizes varied across the included studies, with some having small sample sizes (e.g., *n* = 9–15 per group in Devos et al. [51], Koh et al. [54], and Mitoubsi et al. [55]).

**Table 1:** Characteristics of included studies.

Author	Participants	Modality	Driving Task	Main Neurophysiological Findings
Wascher et al. [56]	395 older (71.4 ± 3.0)	EEG (cEEGrid)	Realistic driving simulation with varying traffic/cognitive load	cEEGrid captured theta/alpha modulation corresponding to mental load during realistic driving in older adults.
Huizeling et al. [52]	17 young (22.9 ± 4.1) 17 older (70.1 ± 5.2)	EEG	Simulated motorway driving with attentional refocusing (braking vs. sign reading)	Older adults showed reduced/delayed frontal theta and posterior alpha modulation during attentional refocusing.
Depestele et al. [49]	27 young (25–35) 27 middle-aged (50–60) 34 older (≥65)	EEG	Lane-keeping task (straight vs. curved segments)	Midfrontal theta upregulation during steering was blunted in older adults; behavioral compensation via reduced speed.
Depestele et al. [50]	27 young (27.2 ± 2.7) 25 middle-aged (55.3 ± 2.9) 29 older (68.8 ± 3.0)	EEG	Lane-keeping with visuocognitive and visuomotor dual-tasks	Reduced midfrontal theta modulation in older adults under dual-task conditions associated with poorer lane-keeping.
Devos et al. [51]	9 with normal cognition (74.2 ± 4.2) 5 with cognitive impairment (69.2 ± 7.8)	EEG	Level 3 automated driving with emergency takeover requests	Cognitively impaired older drivers showed greater frontal theta increase during takeover, with prolonged reaction times.
Mitoubsi et al. [55]	14 controls (74.2) 15 early-stage AD (74.9)	ERP (VEP)	Visual stimulation paradigm + on-road fitness-to-drive assessment	Patients with AD showed delayed/attenuated VEPs; abnormal VEPs predicted unfit-to-drive classifications.
Koh et al. [54]	10 young (22.7 ± 2.9) 10 older (66.2 ± 5.0)	ERP	Traffic sign recognition using simulated HUD	Older drivers showed delayed P300 latency associated with higher error rates in traffic sign recognition.
Karthaus et al. [53]	18 young (21.5 ± 2.3) 18 middle-aged (35.7 ± 2.6) 18 young-old (59.6 ± 3.2) 18 old-old (75.1 ± 2.8)	ERP	Simulated car-following with visual/auditory distractors	Older adults showed reduced P3b amplitude under distraction; the old-old group missed nearly 40% of braking responses.
Eudave et al. [60]	22 young (30.3 ± 4.3) 20 older (67.4 ± 5.2)	fMRI	High-speed visual discrimination (fMRI) + driving simulator	Older adults showed frontoparietal hyperactivation with impaired DMN deactivation; brain–behavior associations present in young were absent in older adults.
Nakata et al. [58]	22 young (21.7) 20 older (70.2)	fNIRS	Simulated driving with red/green light stops	Older adults exhibited left-lateralized prefrontal activation during frustrating red-light stops, correlating with executive function decline.
Kawai & Nakata [57]	21 young (20.6 ± 2.6) 23 older (68.7 ± 2.6)	fNIRS	Bipedal response-position selection task (accelerator–brake)	Older adults showed greater lateral prefrontal activation during complex response selection while maintaining task accuracy.
Stojan & Voelcker-Rehage [59]	37 young (21.7 ± 1.6) 37 older (69.5 ± 3.6)	fNIRS	Simulated car-following with dual-tasks (typing, working memory, argumentation)	Age groups showed different prefrontal activation patterns during dual tasks; prefrontal–performance associations differed by age.

### 3.3. Neurophysiological Findings

#### 3.3.1. EEG Study Findings: Frontal Theta Activity and Executive Function

Four EEG studies investigated midfrontal theta activity during driving simulator tasks [49,50,51,52]. Compared with younger drivers, older drivers consistently exhibited attenuated theta modulation, although specific patterns varied across studies. Depestele et al. [49] observed blunted midfrontal theta upregulation specifically during curved-road steering, with older adults compensating behaviorally through reduced speed. Depestele et al. [50] found that theta dysregulation emerged only under the most demanding dual-task conditions, associated with greater heading error and reduced lane-keeping stability. Huizeling et al. [52] demonstrated reduced and delayed frontal theta and posterior alpha modulation during attentional refocusing between braking and sign reading, with older adults showing larger refocusing costs. Devos et al. [51] further demonstrated that frontal theta dysregulation was exacerbated in cognitively impaired older adults during automated driving takeover, with prolonged reaction times, suggesting a continuum of impairment severity. Compared with younger drivers, older drivers exhibited reduced modulation and diminished theta upregulation, particularly under dual-task conditions and when adapting their actions to changing road conditions. In these executive functioning tasks, older drivers displayed delayed reactions, increased operational errors, and decreased lane-keeping stability. The cEEGrid system effectively captured the real-time cognitive load during driving by detecting increases in theta power and decreases in alpha power from electrodes around the ears, successfully enabling real-time monitoring despite the lack of source localization [56].

#### 3.3.2. ERP Study Findings: Neurophysiological Markers of Attentional Allocation, Driving Responses, and Fitness-to-Drive

Three studies investigated visual processing and attention allocation using event-related potentials [53,54,55]. Older adults showed reduced P3b amplitude in response to visual and auditory distractors during complex braking response tasks, which was associated with longer braking response times and increased error rates. These results indicate that attention is prioritized toward distractors rather than driving-relevant stimuli [53]. In traffic sign recognition tasks, older adults exhibited higher error rates than younger adults, and incorrect responses among older adults were associated with a prolonged P300 latency [54]. Moreover, in patients with AD, delayed latencies and reduced amplitudes of visual evoked potentials (VEPs) in response to visual motion stimuli (optic flow) were associated with unfit-to-drive classifications on on-road tests or validated off-road driving assessments [55].

#### 3.3.3. fNIRS and fMRI Study Findings: Age-Related Activation and Compensation Mechanisms in the Prefrontal Cortex

Three fNIRS studies investigated prefrontal cortex activity during driving-related tasks [57,58,59], whereas one fMRI study examined the dynamics of the DMN during driving [60]. Despite comparable performance in the bipedal/bimanual response-position selection task, older adults demonstrated a pattern of hyperactivation in the lateral prefrontal region compared with younger adults [57]. In addition, in driving tasks involving typing and argumentation, older adults exhibited the most pronounced activation in the prefrontal region during the typing condition, with these activity patterns reflecting different processing strategies compared to those of younger adults [59]. Concurrently, Eudave et al. [60] reported that older adults did not deactivate the DMN and showed hyperactivation in the frontoparietal region during a high-speed visual-discrimination task. The association between DMN dynamics and driving speed observed in young adults was not observed in older adults. Regarding the relationship between emotional regulation and brain activity, Nakata et al. [58] found that only older adults exhibited left-dominant prefrontal activation during repeated red-light stops, which was associated with approach motivation for anger.

## 4. Discussion

### 4.1. Summary of Principal Findings

This scoping review aimed to synthesize neurophysiological evidence on brain activity during driving performance in older adults. We identified 12 studies (EEG/ERP, *n* = 8; fNIRS, *n* = 3; fMRI, *n* = 1) examining neurophysiological characteristics associated with driving abilities in older adults. From this perspective, three neurophysiological characteristics emerge that influence driving performance in older drivers.

First, older adults exhibit age-related declines in cognitive control during steering and dual-task situations. Multiple EEG studies have consistently reported blunted upregulation of midfrontal theta power in response to increased task load in older adults [49,50,51,52,56]. This dysregulation is associated with decreased steering precision, dual-task costs, and delayed attentional switching.

Second, EEG/ERP measures indicate difficulty in allocating attentional resources. ERP studies demonstrated reduced P3b and prolonged P300 latency in older adults [53,54]. In the old-old group, visual distractors in complex conditions led to frequent failures to prioritize braking (nearly 40% missed), along with reduced P3b, indicating unfavorable resource allocation [53]. Furthermore, older adults showed more traffic-sign recognition errors, and incorrect (vs. correct) responses were characterized by delayed P300 latency, suggesting slower stimulus evaluation [54]. In older adults with AD, abnormal VEP to optic flow stimuli—similar to visual information changes experienced during driving—were associated with unfit-to-drive classifications [55].

Third, age-related activation patterns involving cognitive task control and emotional regulation were observed in the prefrontal cortex [57,58,59,60]. fNIRS studies consistently reported that older adults showed stronger prefrontal activation, even when performing tasks at levels comparable to those of younger adults [57,58,59]. An fMRI study also demonstrated impaired DMN deactivation and hyperactivation during a high-speed visual speed discrimination task, with the brain activity–driving speed association observed in young adults being absent in older adults [60]. These findings suggest that older adults mobilize greater neural resources to maintain driving performance. However, whether this hyperactivation truly serves a compensatory function or reflects processing inefficiency remains an open question, discussed later in conjunction with EEG/ERP findings.

Although no formal critical appraisal of methodological quality was conducted, consistent with PRISMA-ScR guidance, several methodological characteristics of the included studies warrant consideration. First, sample sizes varied considerably, ranging from 9 to 395 participants, and many studies employed small samples (*n* < 30), potentially limiting statistical power. Second, the included studies encompassed both EEG/ERP (*n* = 8) and fMRI/fNIRS (*n* = 4) modalities; EEG/ERP directly measures electrical neural activity, whereas fMRI/fNIRS reflects hemodynamic responses. This difference in measurement principles should be considered when integrating findings across modalities. Third, even among EEG/ERP studies, the unit of analysis (continuous driving segments, event-locked epochs, or averaged ERPs), frequency band definitions (fixed bands vs. individualized alpha frequency), and region-of-interest specifications varied across studies, potentially affecting the comparability of spectral power and ERP estimates. Furthermore, ocular artifact correction methods varied across studies (e.g., ICA-based removal, the Gratton–Coles–Donchin procedure), and several studies did not provide explicit descriptions of their ocular artifact handling, which is particularly relevant given the frequent eye movements inherent in driving tasks. Similarly, fNIRS studies varied in the number and spatial coverage of measurement channels—ranging from focused bilateral DLPFC configurations (2 channels) to broader prefrontal arrays (16–18 channels)—as well as in preprocessing pipelines and analytical approaches; notably, none of the included studies reported the use of short-separation channels for systemic physiological noise regression. Fourth, all included studies were conducted using driving simulators or laboratory-based experimental paradigms. The ecological validity of these paradigms differs from that of real-world driving, and this discrepancy should be considered when interpreting brain–behavior associations. These methodological considerations constrain the strength of the mechanistic interpretations derived from this review. Fifth, all included studies employed cross-sectional designs; no longitudinal studies examining the predictive value of neurophysiological markers for future driving decline were identified. This precludes conclusions regarding the prognostic utility of the reported brain–behavior associations.

Given that only 12 studies met the inclusion criteria, the following interpretations should be regarded as preliminary and require corroboration from future research.

### 4.2. Interpretation of Age-Related Neural Changes in the Driving Context

#### 4.2.1. Load-Dependent Dysregulation of Neural Modulation in Older Drivers

Most of the included studies measured neural activity associated with mental workload in driving contexts and during driving events. In this review, we defined “high-load” driving tasks as those involving multiple or compounded cognitive demands, such as driving simulator scenarios with complex traffic situations (e.g., Wascher et al. [56]) or dual-task conditions requiring concurrent performance of driving and secondary tasks (e.g., Stojan & Voelcker-Rehage [59]; Depestele et al. [50]). The minimum shared feature across these high-load conditions is the need to allocate attentional resources concurrently to two or more competing task demands while driving.

In younger adults, increasing task demands during driving elicit a characteristic pattern of frontal theta enhancement coupled with posterior alpha suppression, reflecting engagement of cognitive control processes and adjustments in sensory gating [61,62,63]. Conversely, mind-wandering during driving increases both frontal theta and posterior alpha activity [64,65,66]. This review reveals that such load-dependent oscillatory modulation observed in younger adults is attenuated or disrupted in older adults.

EEG studies consistently report blunted upregulation of midfrontal theta during steering, dual-task performance, and attentional switching [49,50,52]. This dysregulation is further exacerbated in older adults with cognitive impairment, presumably contributing to prolonged takeover reaction times during automated driving [51]. Similarly, ERP studies demonstrate reduced P3b amplitude and prolonged P300 latency during processing of visual distractors and traffic sign recognition [53,54], suggesting impaired attentional resource allocation and delayed working memory updating. Given that attenuated P3b and delayed P300 are established neurophysiological markers of MCI and AD [67], abnormal visual evoked potentials to optic flow stimuli associated with unfit-to-drive classifications in patients with AD [55] suggest that impaired responses to visual motion stimuli and their cognitive processing characterize older drivers with cognitive decline.

In general, older adults do not exhibit the task-demand-dependent increase in theta power typically observed in younger adults [32,33]. Posterior alpha power also decreases with age [34,36], and either remains unchanged or paradoxically increases under cognitively demanding tasks [34,35]. Although the resting-state theta/alpha ratio increases in older adults, particularly those with MCI or AD (EEG slowing), task-related theta/alpha modulation is reduced or less pronounced [67]. Therefore, while younger adults allocate neural resources to the prefrontal cortex in response to driving task demands, older adults show reduced capacity for such switching, which may result in driving behavior instability and errors, such as missing traffic signs under interference or increased task load.

Critically, the behavioral consequences of this neural dysregulation are not uniform but emerge most prominently under high-load conditions. Although older drivers maintain adequate performance in simple scenarios through conservative strategies such as speed reduction [49], such compensation fails under high-demand conditions characterized by complex distractors, rapid switching, and dual-task cognitive demands. In the old-old group (mean age: 75.1 ± 2.8) studied by Karthaus et al. [53], approximately 40% of braking responses were omitted under visual distraction, consistent with reduced P3b amplitude. Similarly, Depestele et al. [50] reported disrupted dual-task-related theta modulation only under the most challenging conditions, leading to lane-keeping failures. These findings suggest that age-related neural changes increase vulnerability specifically under high cognitive load rather than causing generalized driving impairment. Real-time monitoring using the cEEGrid system has demonstrated the feasibility of capturing such theta/alpha power modulation during actual driving [56].

#### 4.2.2. Prefrontal Hyperactivation: Compensation and Its Limitations

fNIRS studies in younger drivers show increased prefrontal cortex activation during high mental workload while driving [68,69] and a relative reduction in posterior activity owing to distracting stimuli [70]. These findings suggest that while information processing and updating in prefrontal working memory enable complex driving operations, allocating attentional resources to distracting stimuli increases workload, potentially leading to driver fatigue, reduced attention, and impaired visuospatial processing, as frontal resources are concentrated.

The fMRI and fNIRS studies in this review consistently report prefrontal hyperactivation in older adults during driving tasks [57,58,59,60]. Although not captured by our search strategy, a related fMRI study by Eudave et al. [71] similarly reported prefrontal and parietal hyperactivation with increased frontoparietal connectivity in older adults during an egocentric distance perception task relevant to driving, further supporting the generalizability of this age-related neural pattern. Within the Scaffolding Theory of Aging and Cognition Revised (STAC-r) framework [72], this hyperactivation may reflect compensatory recruitment of additional neural circuits to offset declining neural processing efficiency and resource allocation, as evidenced by EEG/ERP studies. Indeed, Kawai & Nakata [57] demonstrated that older adults maintained pedal response accuracy despite increased lateral prefrontal recruitment, consistent with effective compensation under moderate task demands. The distinct prefrontal activation patterns observed in older versus younger drivers during car-following with dual-task demands [59] also suggest compensatory neural processing strategies.

However, the STAC-r model posits that as task load increases, the neural “scaffolding” reaches its limits and performance declines; indeed, hyperactivation under high task demands may impair performance [41,73]. For successful task performance, older adults show increased functional connectivity and reorganization among the prefrontal cortex, parietal lobe, and attention networks [39,40]. Notably, the DMN exhibits reduced suppression and connectivity dissociation, which is associated with poor cognitive control and attention function [37,41]. Thus, the dissociation between neural effort and behavioral outcome may depend on the integrity of network-level coordination. Eudave et al. [60] reported that older adults exhibited frontoparietal hyperactivation, alongside impaired DMN deactivation and reduced within-DMN connectivity. Notably, brain-behavior associations observed in younger adults—linking DMN dynamics to driving speed and frontoparietal activation to task accuracy—were absent in older adults. This pattern suggests that when task-mode switching (i.e., DMN suppression) fails, additional frontal recruitment may represent inefficient rather than compensatory processing. Similarly, the high frustration and prefrontal hyperactivation associated with frequent red-light stops during driving suggest additional prefrontal resource recruitment for emotional inhibition and control, which may consequently reduce prefrontal resources available for complex task processing and rapid responses during driving [58].

Integrating hemodynamic and EEG/ERP findings, we hypothesize that the efficiency of prefrontal compensation in the driving context for older adults depends on the preservation of task-related neural oscillatory modulation. When midfrontal theta upregulation and posterior alpha suppression remain intact, prefrontal hyperactivation may effectively support driving performance. Conversely, a disrupted neural oscillatory pattern in the EEG under high-load conditions, in the absence of upregulation of midfrontal theta, suggests recruitment of frontal resources without the coordinated oscillatory state required for effective executive function regulation. This mismatch may lead to additional or compensatory non-functional recruitment of frontal resources, or to depletion of neural resources due to limited prefrontal capacity under emotional load. In such cases, increased variability in driving behavior, reduced safety margins, and elevated error rates may ensue.

Prefrontal hyperactivation in older adults should be interpreted in the context of task demand and behavioral performance. When performance is preserved under relatively mild-to-moderate load conditions, additional prefrontal recruitment may reflect successful compensation. For example, increased lateral prefrontal activation accompanied by preserved pedal response accuracy suggests that compensatory recruitment can provide functional benefit under manageable task demands [57]. In contrast, under high-load conditions, such as dual-tasking [50], visual distractors [53], rapid attentional switching [52], or complex traffic environments [56], this additional recruitment may no longer provide sufficient functional benefit and may instead reflect inefficient neural recruitment or compensatory failure. Therefore, prefrontal hyperactivation should not be interpreted as a uniformly positive marker, but rather in relation to task demand and the associated behavioral adaptation. However, the point at which successful compensation shifts to compensatory failure cannot yet be determined quantitatively, because it is likely influenced by both task design and substantial inter-individual variability.

Notably, this mechanistic account remains speculative within the limited evidence base of this review; no included study employed simultaneous EEG and hemodynamic imaging, and variability in signal processing approaches—including ocular artifact correction in EEG studies and the absence of short-separation channel regression in fNIRS studies—may affect the reliability of the reported neural activation patterns on which this framework is based. The proposed framework should therefore be considered hypothesis-generating. Verification requires multimodal studies combining EEG with fNIRS or fMRI during ecologically valid driving tasks. Furthermore, the experimental paradigms of the included studies can be broadly categorized into driving simulator tasks that replicated naturalistic driving contexts [49,50,51,52,53,56,58,59] and laboratory-based tasks that either isolated specific cognitive or response components [54,57] or examined indirect associations between driving behavior and brain function [55,60]. Although all these paradigms assessed driving-related behavior, cognitive function, or cognitive processes, direct comparison of findings across these different paradigms and interpretation of their correspondence to real-world driving behavior and driving ability should be made with caution. Nevertheless, the convergence of findings across these diverse paradigms—particularly the consistent observation of blunted midfrontal theta modulation and prefrontal hyperactivation in older adults—supports the robustness of the overall patterns identified in this review.

#### 4.2.3. Clinical Implications: A Load-Dependent Risk Model

Driving is a complex task requiring coordinated physical and mental functions. Driving tasks involving visual and auditory stimuli (and occasionally somatosensory input) are continuous, variable, and sometimes simultaneous, demanding diverse cognitive processing based on prediction. The convergent findings of this review indicate that, under mild to moderate driving load conditions, older adults maintain driving performance through cognitive and behavioral compensatory strategies, whereas under high-load conditions, the breakdown of neural compensation leads to marked performance decline. Accordingly, prefrontal hyperactivation in older adults should not be regarded as a uniformly positive marker in fitness-to-drive assessment. Rather, its clinical significance should be interpreted in relation to task demand, behavioral performance, and complementary neurophysiological indicators. Hyperactivation observed under mild-to-moderate load may reflect successful compensation, whereas similar activation under high-load conditions may indicate emerging vulnerability or a breakdown of compensatory processes. Based on this load-dependent risk model, the following key points for neurophysiological driving fitness assessment are proposed.

First, driving fitness assessments should incorporate high mental load scenarios—including dual-task conditions, complex distractors, and rapid switching demands—to unmask vulnerabilities not apparent under routine driving conditions [50,53]. Second, visual processing integrity warrants specific evaluation; the association between abnormal VEPs to optic flow stimuli and unfit-to-drive classifications [55] suggests that upstream perceptual deficits may cascade into downstream decision-making failures. Third, wearable EEG systems [56] offer the potential for practical neurophysiological monitoring and real-time detection of cognitive overload during actual driving. Fourth, further research is needed to clarify the contribution of emotional factors; the left-lateralized prefrontal response to frustrating traffic situations in older adults [58] indicates that emotional load compounds cognitive demands and may contribute to risky behavior. Since the left prefrontal cortex plays a key role in dual-task performance in older adults [74], the need for frustration regulation while driving might interfere with complex driving task performance.

These findings are particularly significant for clinical populations. Theta dysregulation and prolonged takeover times observed in older drivers with cognitive impairments [51], and VEP abnormalities in AD drivers [55], suggest that neurophysiological markers may serve as objective, non-invasive biomarkers for driving fitness evaluation and possibly complement traditional cognitive testing and on-road assessment. However, current clinical fitness-to-drive assessments rely primarily on neuropsychological testing and on-road evaluations, and a substantial gap remains—including engineering, regulatory, and practical barriers—between the specialized experimental conditions under which these findings were obtained (including research-grade neurophysiological recording and analysis systems and driving simulators capable of implementing cognitively demanding scenarios) and routine clinical practice. With this gap in mind, future research should determine optimal thresholds and combinations of these markers for fitness-to-drive evaluation and explore whether targeted cognitive or perceptual interventions significantly affect the development of sufficient driving skills and neural plasticity to support safe driving in realistic driving situations.

### 4.3. Limitations of This Review

This review has some limitations. First, it was restricted to English-language literature, potentially excluding relevant papers published in other languages. Second, Web of Science was not searched because of institutional access restrictions. Although Scopus was included to complement the interdisciplinary scope, relevant literature may have been missed. Third, consistent with PRISMA-ScR guidance, no formal critical appraisal of risk of bias or methodological quality was conducted. The included studies also showed variability in sample sizes and methodological heterogeneity, including differences in data processing and analytic pipelines. Fourth, as only 12 studies were included, modality-specific bias emerged (i.e., EEG studies comprised two-thirds of the included literature), which constrains the reliability of our mechanistic interpretations presented in this review. However, considering methodological challenges in measurement, this bias may reflect the advantages of EEG measurement in neurophysiological assessment of driving. Fifth, our restriction of the search period to 2015–2025 may have excluded earlier neurophysiological studies on driving in older adults. However, because many of these earlier studies employed data analysis pipelines that differ from those currently considered standard, direct integration of their findings with contemporary evidence would be methodologically challenging. Sixth, although all older adult groups in the included studies had a mean age of 65 years or older, consistent with our inclusion criterion of ≥60 years, only Karthaus et al. [53] included a subgroup centered around age 75 years. Furthermore, studies involving older adults with cognitive impairment were limited to Devos et al. [51] and Mitoubsi et al. [55]. Therefore, the generalizability of our review findings to drivers aged 75 years and older and to those with cognitive impairment remains limited. Seventh, none of the included studies reported sex-stratified neurophysiological findings. Given that sex differences in cognitive aging trajectories and prefrontal compensatory patterns have been reported [75,76], these differences may differentially influence the neurophysiological underpinnings of driving performance in older adults. Future research should consider sex as a variable in neurophysiological studies of driving. Eighth, as detailed in Table S2, only one study reported classification accuracy for categorical driving outcomes using machine learning, and no study established diagnostic cutoff values for individual neurophysiological markers. The brain–behavior correlations reported across studies were generally small to moderate in magnitude, and most did not survive correction for multiple comparisons. These findings highlight the current lack of evidence for the diagnostic utility of neurophysiological markers in driving fitness evaluation and underscore the preliminary nature of the conclusions drawn in this review. Finally, because task paradigms and operational definitions of “high-load” conditions differed across studies, the point at which compensation breaks down could not be quantified and is likely to vary substantially across individuals.

## 5. Conclusions and Future Directions

This review suggests that older drivers exhibit blunted load-dependent theta modulation, declines in attention-related ERP components, and prefrontal hyperactivation, which may reflect compensation under manageable demands but vulnerability under high-load conditions. These patterns indicate that age-related neural changes in driving are load-dependent and become behaviorally relevant, particularly in cognitively demanding situations. Based on this review, we propose several directions for future research.

First, studies should target older adults with adequate sample sizes, particularly those aged 75 years and older and those with cognitive impairment who are at higher risk of driving. Neurophysiological studies of driving frequently use driving simulators owing to constraints on the measurement environment, which are prone to inducing motion sickness in older adults [77]. Consequently, participant recruitment tends to be biased toward younger older subjects, necessitating adjustment of task scenarios and driving duration to accommodate the driving tolerance of older drivers.

Second, there is scope to examine correlations between on-road assessment and neurophysiological markers. Wearable EEG systems extend the feasibility of EEG measurement during actual driving, and visual- and attention-related ERPs from laboratory experiments may serve as predictors of on-road driving fitness.

Third, long-term, prospective neurophysiological studies can shed light on how pathological changes that occur as older adults progress from being cognitively normal to experiencing cognitive decline are connected to neurophysiological alterations and decreases in driving ability. This finding may also contribute to the assessment of driving and dementia risk. In this regard, cross-sectional studies targeting older adults with subjective cognitive decline or subjective memory complaints, as well as those with MCI or dementia, will be needed to clarify the relationship between cognitive impairment and driving risk in older adults.

Fourth, future research should clarify the role of prefrontal hyperactivation during demanding cognitive driving tasks. It remains to be verified whether the upward hemodynamic changes in the frontal region identified in this review truly compensate for diminished synchronous neural oscillations or result in redundant neural recruitment that does not affect performance. This finding requires verification through multimodal neurophysiological studies that employ simultaneous EEG and fNIRS/fMRI recordings.

Finally, although older adults may perform comparably to younger drivers under relatively mild cognitive demands in driving, age-specific neurophysiological patterns may already be present in the background. Future research should determine whether such latent neural patterns can predict critical driving errors or fitness-to-drive classifications before overt behavioral decline becomes apparent. To achieve this, studies should establish standardized driving tasks and outcome definitions, and identify clinically meaningful thresholds for neurophysiological markers, whether based on single markers or combinations of markers, within standardized task paradigms. These thresholds should be validated against simulator- or on-road-based fitness-to-drive outcomes with clinically acceptable accuracy.

## Data Availability

The protocol for this scoping review was registered on the Open Science Framework (OSF) [https://doi.org/10.17605/OSF.IO/KA5G4]. The detailed data extraction table is available as Table S2: Detailed characteristics of included studies.

## Supplementary Materials

The following supporting information can be downloaded at: https://www.mdpi.com/article/10.3390/jcm15082956/s1, Table S1: PRISMA-ScR checklist; Table S2: Detailed characteristics of included studies.

## Abbreviations

The following abbreviations are used in this manuscript:

ADAS	Advanced driver-assistance systems
MCI	Mild cognitive impairment
EEG	Electroencephalography
fMRI	Functional magnetic resonance imaging
DMN	Default mode network
fNIRS	Functional near-infrared spectroscopy
PET	Positron emission tomography
ERP	Event-related potential
VEPs	Visual evoked potentials
